# Technical insights and implications of thoracolumbar spine and neural tissue harvesting in recently deceased organ donors: a direct anterior approach integrated into multi-organ procurement protocols

**DOI:** 10.1007/s00586-025-09675-2

**Published:** 2025-12-26

**Authors:** Paolo Brigato, Federico Cardahi, Kai Sheng, Hosni Cherif, Li Li, Christopher Coluni, Kirby Upshaw, Jean Albert Ouellet, Lisbet Haglund

**Affiliations:** 1 Research Unit of Orthopaedic and Trauma Surgery, Department of Medicine and Surgery, Università Campus Bio-Medico di Roma, Rome, Italy; 2 Fondazione Policlinico Campus Bio-Medico di Roma, Rome, Italy; 3 McGill University, Department of Surgery, Montreal, Canada

**Keywords:** Organ Donor Model, Spine Harvest, Spine Research, Thoracolumbar Spine, Human cadaveric spine, Intervertebral Disc, Degeneration

## Abstract

**Purpose:**

To present a novel anterior surgical technique for harvesting thoracolumbar spinal tissue from cadaveric organ donors. The approach aims to ensure biological viability of harvested spinal tissue, while maintaining full compatibility with routine multi-organ procurement protocols. By addressing the limited availability of anterior harvesting methods, this technique aims to expand opportunities for high-quality experimental and translational spine research.

**Methods:**

A direct anterior retroperitoneal approach was employed to harvest thoracolumbar spinal columns en bloc, including the spinal cord, dorsal root ganglia (DRGs), vertebral bodies, intervertebral discs, facet joints, and posterior elements. All extractions were performed within 1–2 h following aortic cross-clamping to minimize ischemic time. The surgical procedure included targeted osteotomies to preserve structural continuity, with intraoperative radiographic imaging performed to ensure alignment and suitability for subsequent research use. Tissue samples underwent same-day processing for experimental use, including viability assays to assess the cellular health of key structures.

**Results:**

This harvesting protocol was applied in over 340 cadaveric donors as part of a high-volume organ donation program. The mean harvesting time was approximately 30 min. In all cases, structural continuity of the anterior spinal column was preserved, with radiographic imaging confirming appropriate alignment and completeness of the harvested specimens. Cell viability assessments demonstrated excellent preservation of biologically active components, including viable disc and joint tissues, neural structures, and resident cell populations. The harvested tissues have been successfully used in a variety of research projects.

**Conclusion:**

This anterior spinal harvesting technique is a safe, efficient, and highly reproducible method that can be seamlessly integrated into standard multi-organ procurement workflows. It enables the acquisition of sterile, anatomically intact, and biologically viable spinal tissues from cadaveric organ donors without compromising donor reconstruction or surgical logistics. This approach substantially enhances the availability and quality of human spinal specimens for research and may serve as a useful model for tissue recovery in the field of spine science.

## Introduction

In recent years, the study of degenerative diseases associated with the intervertebral discs (IVDs) and posterior spinal components has led to a growing demand for spinal column specimens [1–5]. Studying human spinal columns offers distinct advantages over using animal-derived specimens [6–8]. Over the years, several techniques have been developed to harvest spinal columns from cadaveric donors, aiming to obtain specimens suitable for a variety of anatomical and research applications [9]. Most of the described anterior approaches have been focused on the retrieval of the cervical spine. In 1984, Rodionov et al. introduced a technique for rapidly removing the vertebral column during autopsy by detaching the cervical spine from the skull base and extracting it anteriorly while preserving the atlanto-occipital joint [10]. This method was refined in 1998, when an anterior approach was employed to extract the intact cervical spine through an opening at the base of the skull for forensic purposes, and in the same year, another technique was described for harvesting the lower cervical spine (C2–C7) using an anterior approach [11].

In contrast, surgical techniques for the thoracolumbar segment have primarily been limited to posterior approaches [12]. The human anterior spinal column represents a valuable resource for surgical innovation, disc degeneration research, tissue regeneration, biomaterial development, and the study of bone–disc interactions in spinal pathologies. It also serves as a fundamental model for preclinical testing, neuroanatomical investigations, and basic research into the mechanisms of IVD degeneration and treatment response [13–15]. The ability to harvest the anterior spinal column with the donor positioned supine further simplifies the procedure, obviating the need to reposition the patient, an essential consideration when organ harvesting for transplantation has already been performed. Furthermore, the ability to easily and comprehensively harvest neural tissues from donors enables targeted studies on the role of the spinal cord and dorsal root ganglia (DRGs) in pain transmission and supports the development of novel therapeutic strategies for chronic spinal pain [16, 17].

The present article describes an innovative surgical technique for harvesting the thoracolumbar spinal column, along with the relevant nerve tissues, without the need for additional surgical incisions beyond those typically required for organ harvesting. This procedure is seamlessly integrated into the standard multi-organ procurement workflow in recently deceased organ donors, ensuring that the harvested tissues are fresh, sterile, and biologically viable.

## Harvesting technique and cell isolation protocols

The technique described in this article was developed and implemented with informed consent and in collaboration with the regional organ procurement organization. Ethical approval for the study was obtained from the Institutional Review Board (IRB #2019–4896) of the host institution. This partnership has played a pivotal role in facilitating the integration of spinal tissue harvesting into the existing organ donation workflow, ensuring that the procedure adheres to the highest ethical and procedural standards.

### Inclusion and exclusion criteria

Harvesting is approved by the regional organ procurement organization, which contacts the research team when an available organ donation is identified. All necessary consents for research are obtained from the patient’s family in accordance with their policies. Inclusion criteria apply to donors of all ages, genders, and ethnic backgrounds. Exclusion criteria include donors with a history of cancer, radiation therapy, Human Immunodeficiency Virus (HIV), or Hepatitis B positive serology.

### Organ donor positioning and dissection

The cadaver is positioned supine on the surgical bed. A midline anterior skin incision is made, extending from the suprasternal manubrium (approximately at the T1 level) down to the level of S1. This exposure allows entry into both the thoracic and abdominal cavities. To facilitate this, a full anterior thoracotomy and laparotomy are performed. At this stage, a standard multi-organ procurement procedure may be carried out, including removal of the heart, lungs, liver, pancreas, and kidneys.

**Fig. 1:**
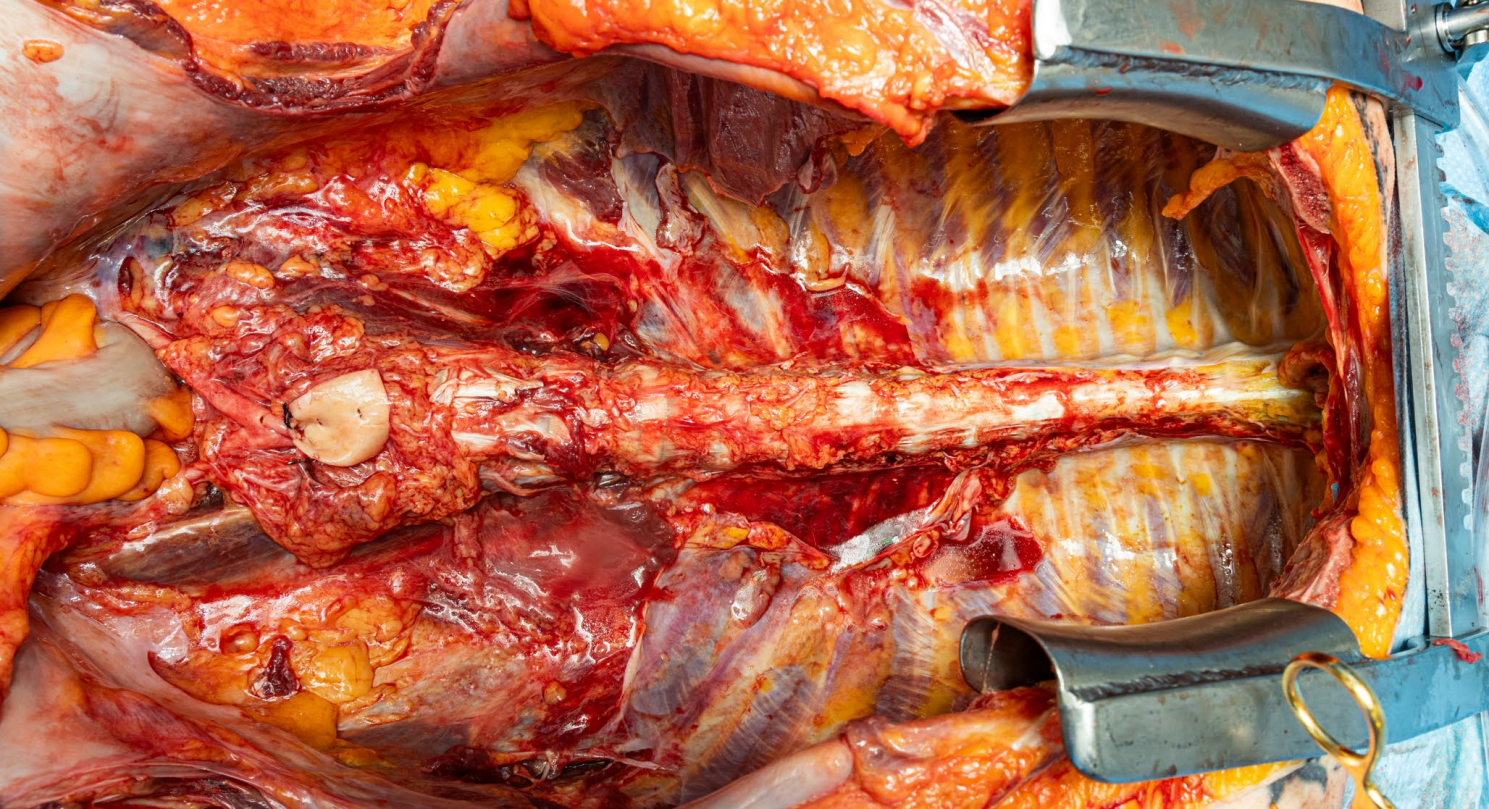
Anterior exposure of the thoracolumbar spinal column

The spinal procedure begins following completion of the organ harvest, typically 1–2 h after cross-clamping, depending on the number of organs retrieved. For the lumbar spine, an anterior intraperitoneal approach is employed: the abdominal contents are gently mobilized and retracted to expose the anterior lumbar vertebrae. For the thoracic spine, a direct anterior transthoracic approach is performed, requiring entry into the thoracic cavity to access the mid and upper thoracic vertebrae (Fig. 1).

**Fig. 2:**
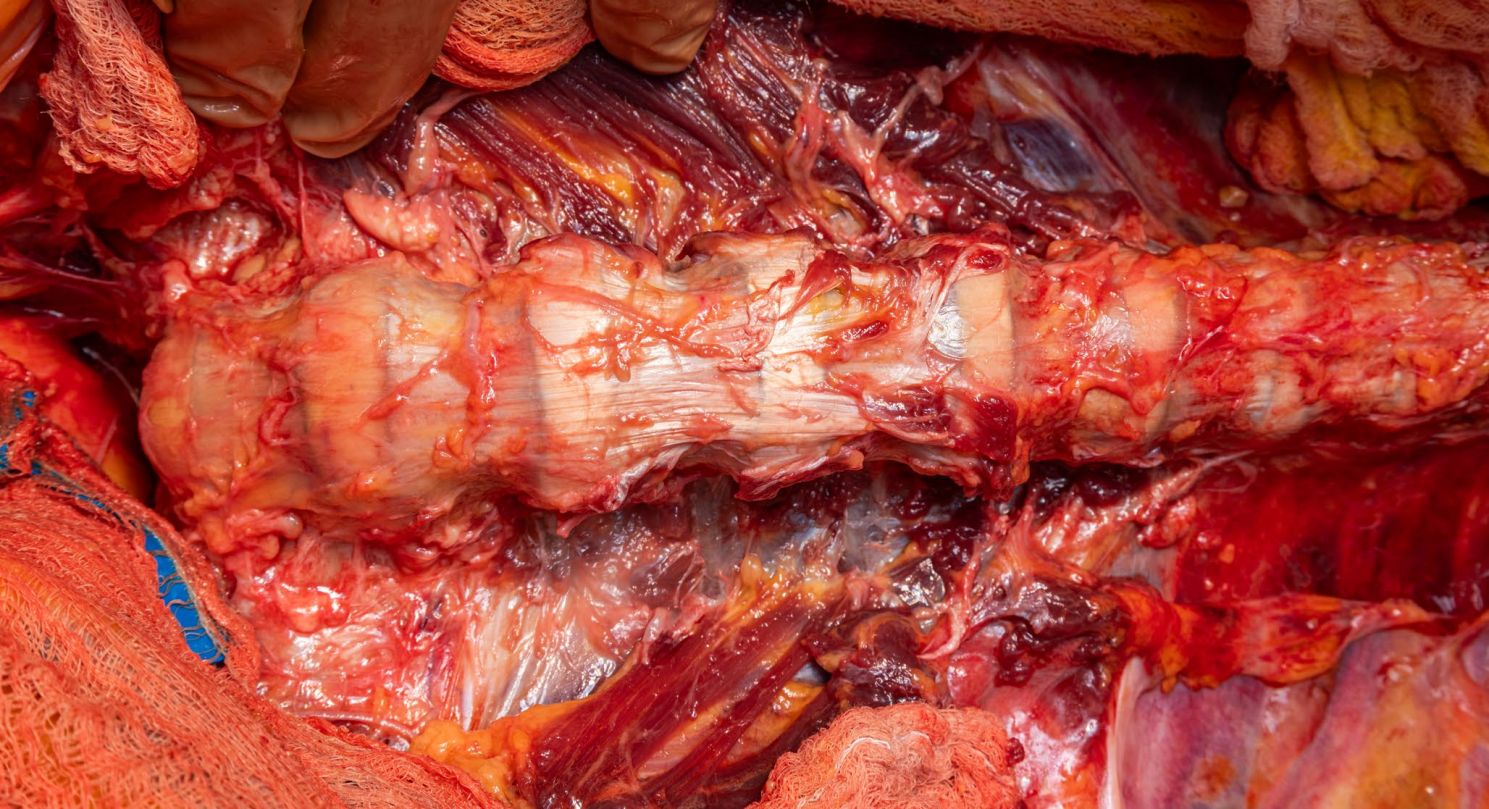
Detachment of the psoas muscles and anatomical exposure of vertebral landmarks

Soft tissue dissection is performed with a No. 23 scalpel and/or Metzenbaum scissors, carefully detaching the psoas major (and minor, if present) from its attachments to the vertebral bodies and transverse processes of the lumbar vertebrae. For more precise and controlled dissection, the Metzenbaum scissors are employed from the lateral side of the vertebral body toward the medial portion of the psoas muscle. This technique facilitates safer tissue release and improves visualization, ultimately providing complete exposure of the lateral aspect of the vertebral body, pedicles, and transverse processes. (Fig. 2).

**Fig. 3:**
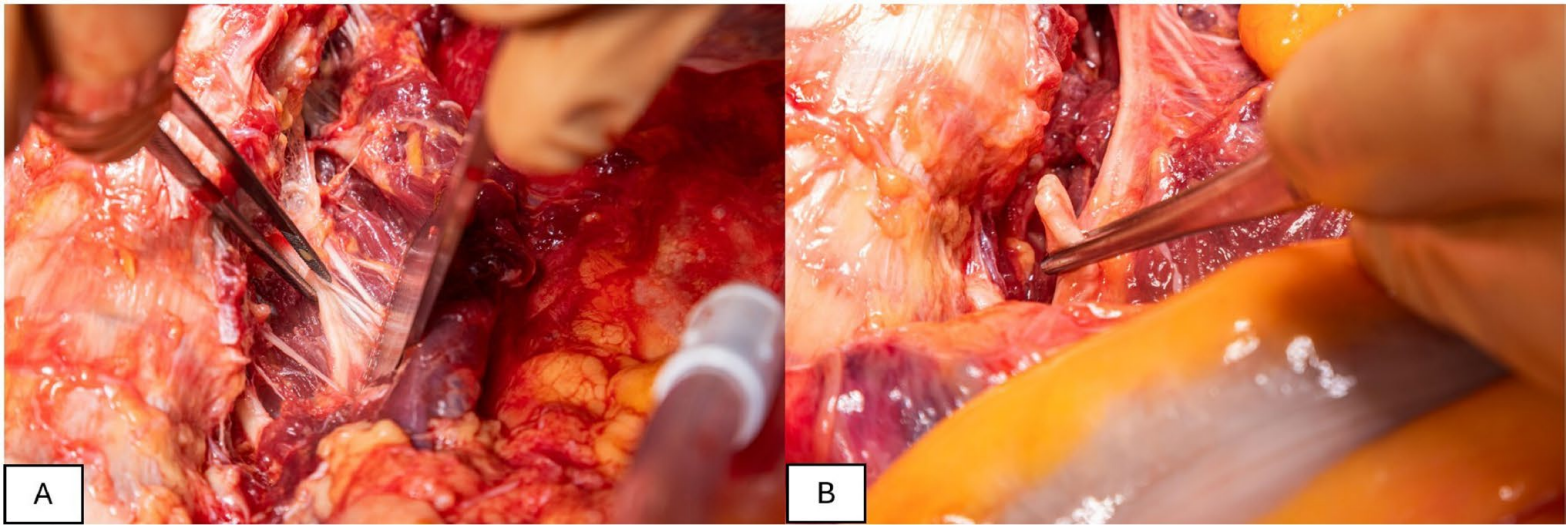
(**A**) Identification and isolation of exiting spinal nerve roots at the neural foramina. (**B**) Transection of the nerve roots approximately 2 cm distal to the foramina

During the psoas muscle detachment procedure, the exiting spinal nerve roots are bilaterally identified, isolated, and transected approximately 2 cm distal to their respective foramina (Fig. 3). This step is crucial, as the remaining proximal nerve segment serves as an anatomical guide for the subsequent identification and dissection of the DRGs.

### Spinal detachment technique

**Fig. 4:**
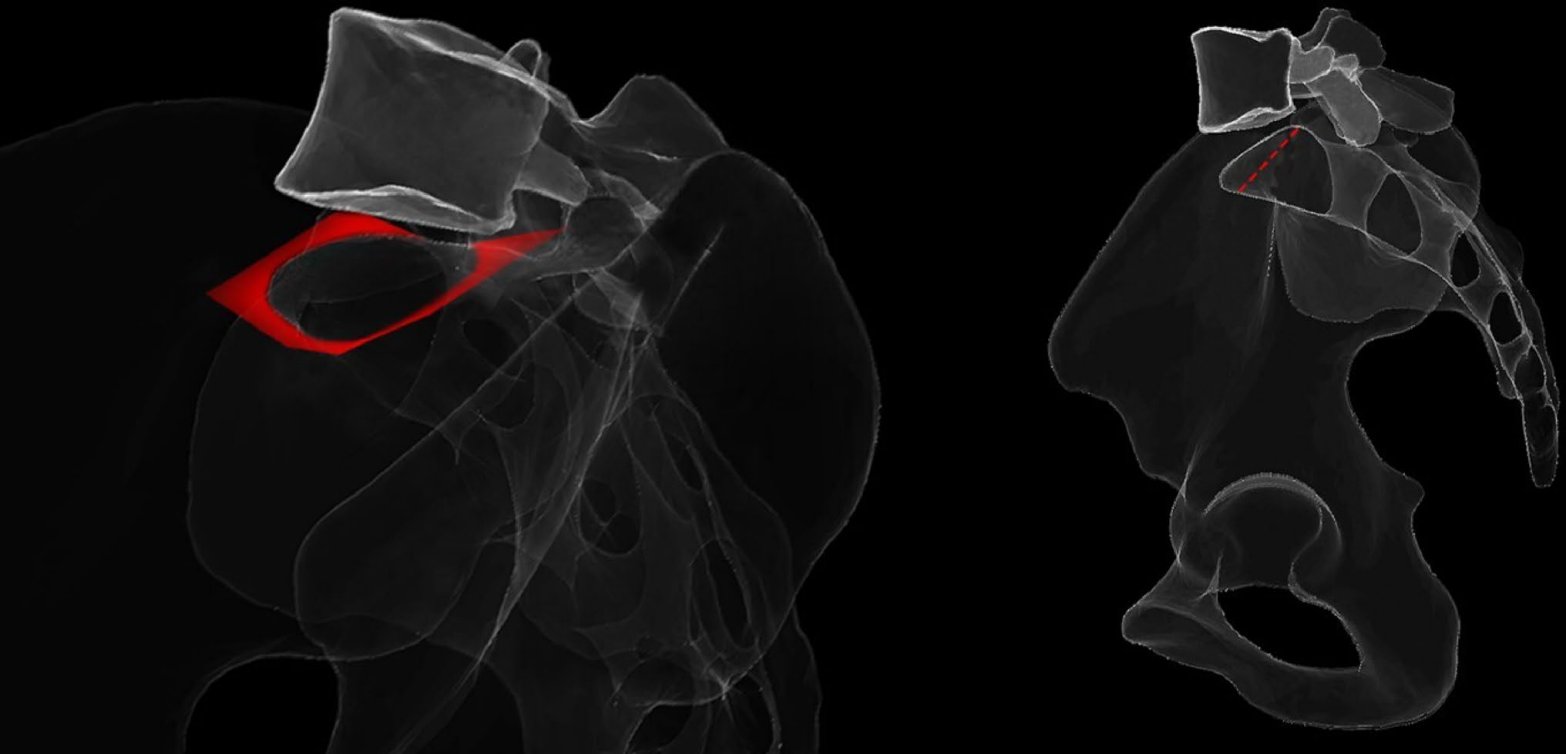
Graphic representation of sacral osteotomy

Inferior detachment of the spine is initiated at the sacral level. Using curved bone osteotomes—or a surgical saw, if available—a U-shaped osteotomy is performed bilaterally through the sacral alae. The horizontal portion of the osteotomy is made in the midline between the sacral promontory and the anterior sacral foramina, effectively separating the caudal end of the spine (Fig. 4).

**Fig. 5:**
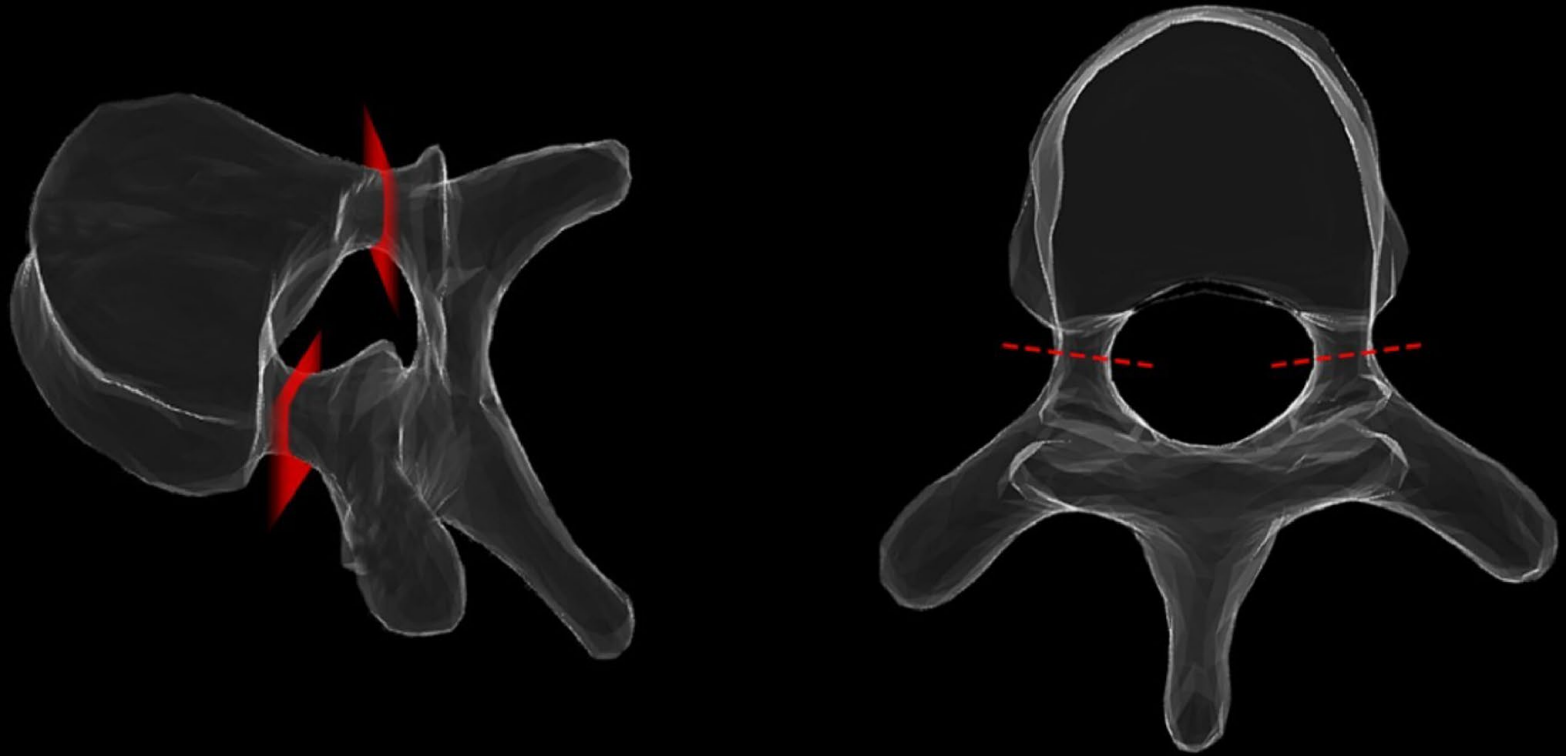
Graphic representation of pedicle osteotomy

Next, the vertebral bodies are bilaterally separated from their posterior elements. This is achieved by inserting a curved osteotome with its bladed corner positioned within the intervertebral foramen and performing a transverse osteotomy at the pedicle midline, repeating the maneuver at each vertebral level planned for harvesting (Fig. 5).

**Fig. 6:**
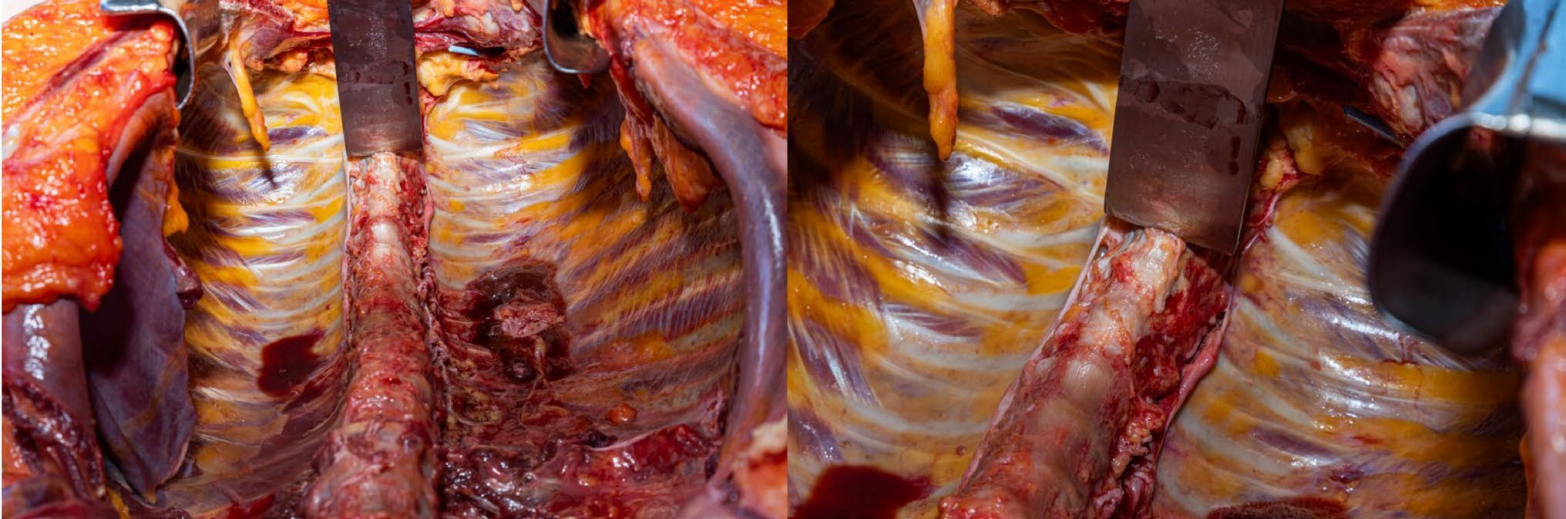
Cranial detachment of the anterior spinal column via a 90° osteotomy

Cranial detachment is then performed using a straight osteotome to execute a perpendicular (90°) osteotomy through the selected vertebral body (Fig. 6). Care is taken to preserve the adjacent IVD spaces during this step.

### Anterior spinal column and neural harvesting

**Fig. 7:**
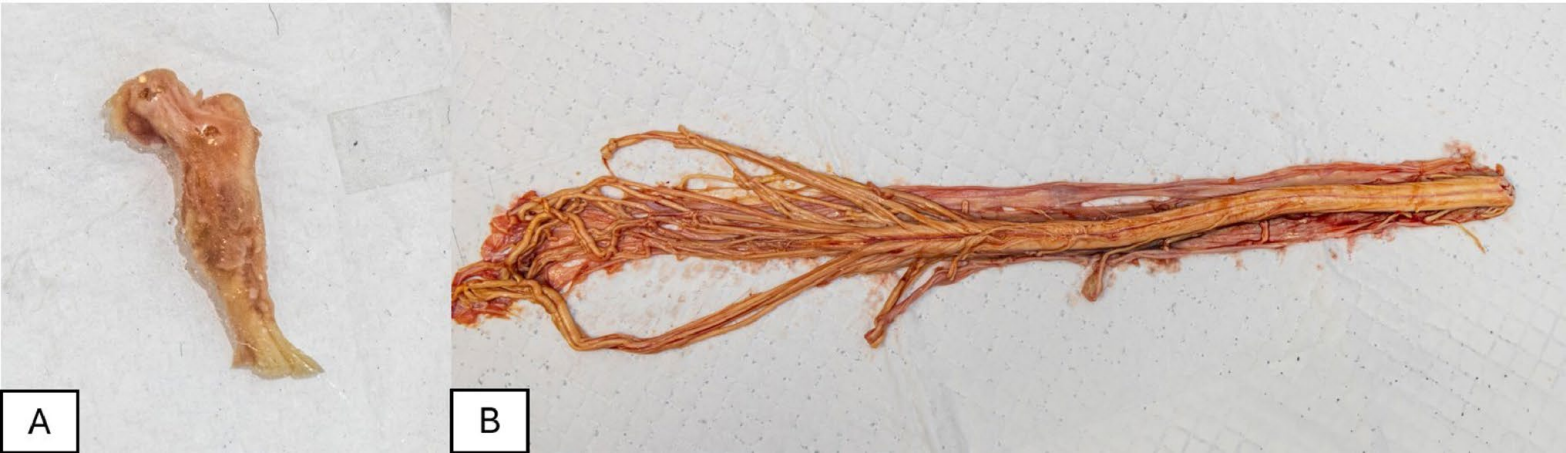
(**A**) Harvesting of dorsal root ganglia (DRGs). (**B**) Harvesting of the spinal cord

**Fig. 8:**
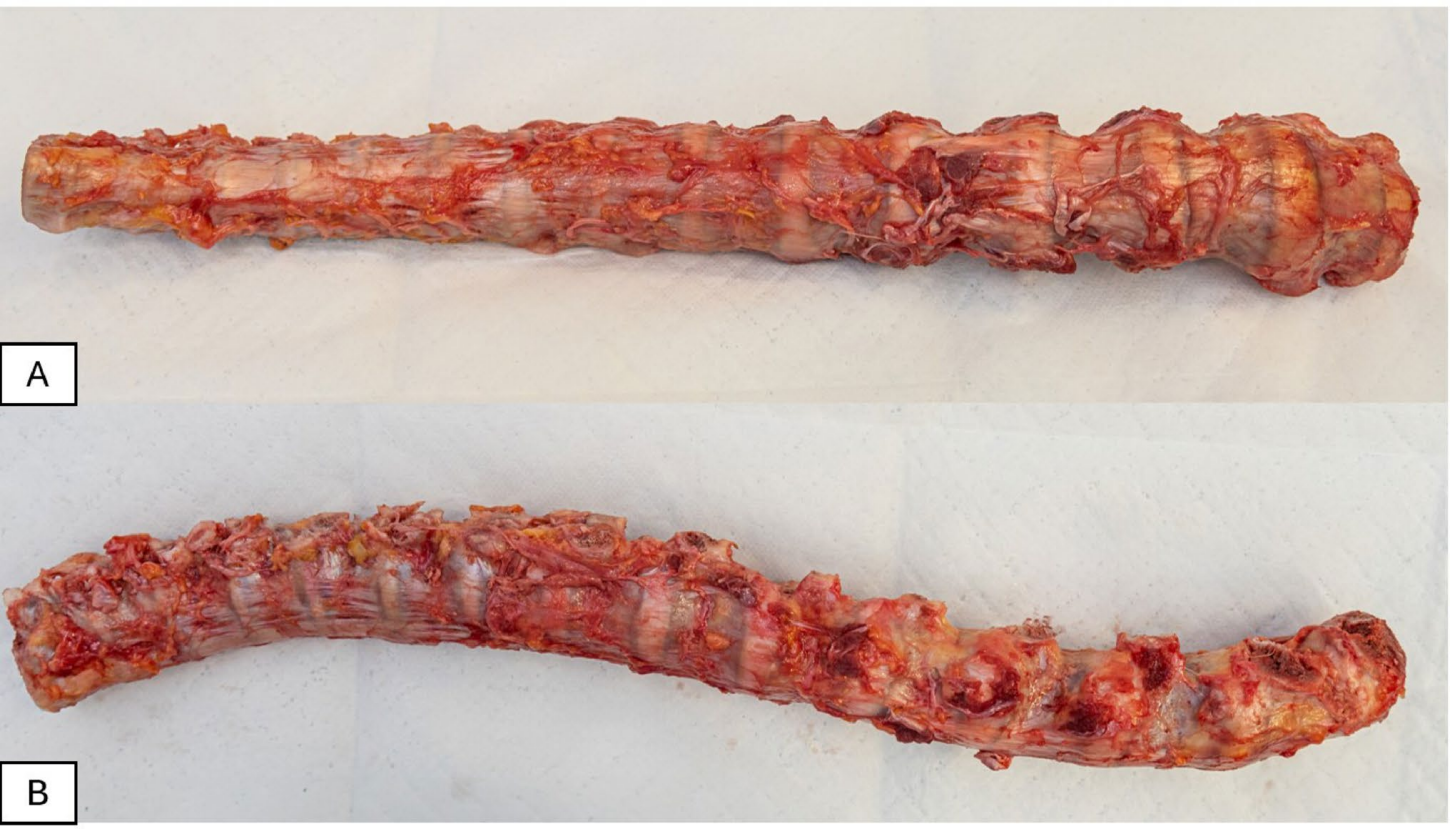
(**A**) Antero-posterior and (**B**) lateral view of the harvested anterior spinal column

Once the osteotomies are completed, the anterior spinal column can be mobilized, providing direct access to the spinal canal and its contents, including the spinal cord, conus medullaris, and filum terminale. A No. 11 surgical blade is then used to transect the spinal cord at the preselected upper level. During this dissection, any fibrous attachments to the dura mater should be carefully released to prevent tearing. The DRGs, located within the intervertebral foramina, can be identified by their distinctive fibrous texture upon palpation. These structures are dissected and transected both proximally and distally to allow for en bloc harvesting, with final transection of the dura mater at the L5–S1 level, completing the removal of the spinal cord (Fig. 7), along with the anterior spinal column (Fig. 8).

### Posterior spinal column harvesting

**Fig. 9:**
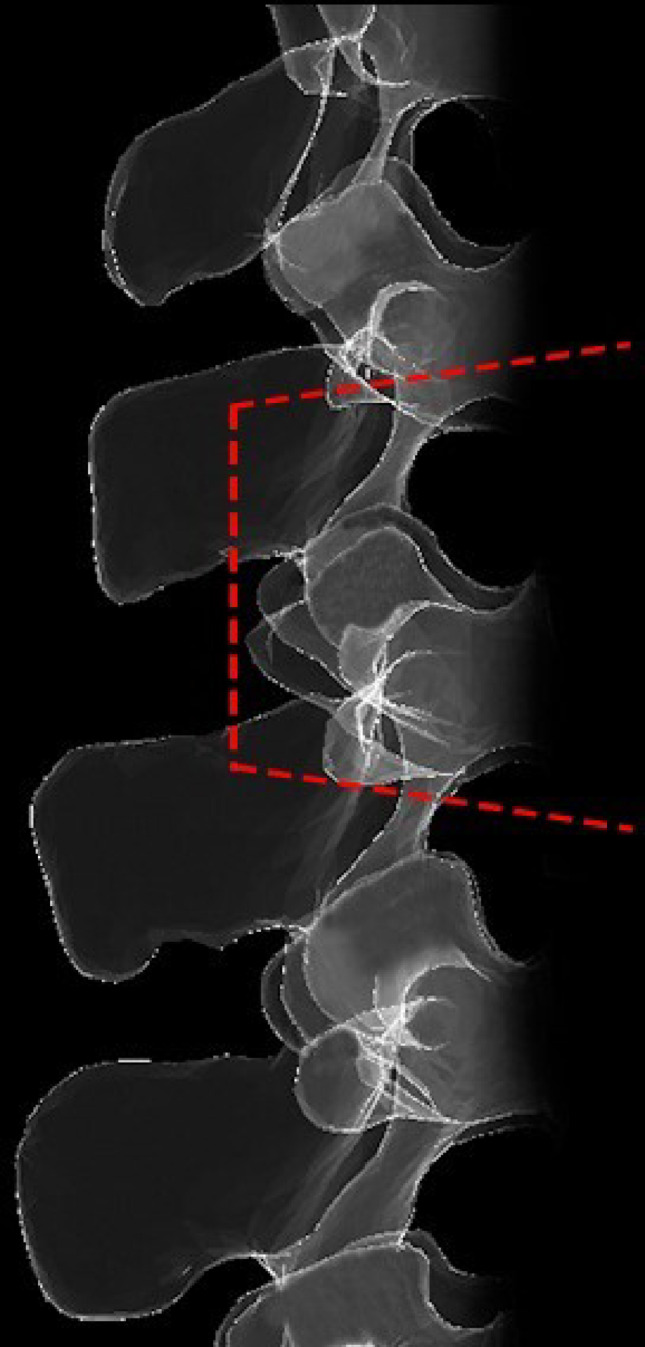
Graphic representation of posterior column osteotomy

The harvesting of the posterior elements begins with careful dissection of the posterior muscles along the transverse processes. Next, two precise inclined osteotomies are performed above and below the facet joint. After elevating the bony structure toward the contralateral side, a midline osteotomy is performed through the spinous process, ensuring preservation of the interspinous ligament (Fig. 9).

**Fig. 10:**
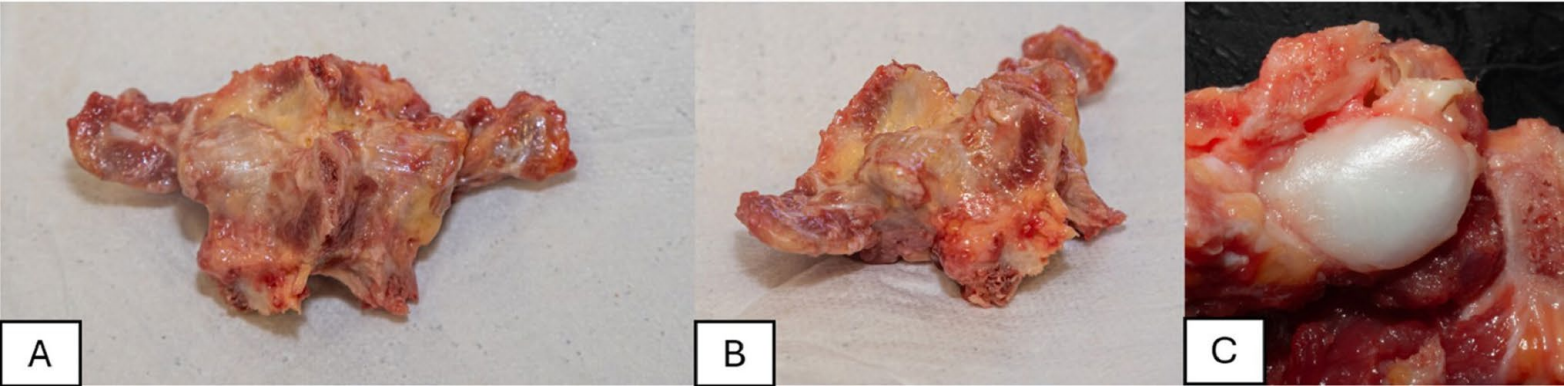
(**A**, **B**) Posterior spinal column specimen harvested for cellular applications. (**C**) Intact facet joint opened under sterile conditions to obtain viable cartilage cells

The facet joints are harvested with the capsule intact and opened under a sterile culture hood to ensure aseptic conditions during tissue processing (Fig. 10). This procedure involves harvesting bilateral facet joint pairs from non-consecutive vertebral levels, which can be used as control samples for comparison with pathological tissues, such as those from patients with adolescent idiopathic scoliosis (AIS), adult degenerative scoliosis, or other facet joint–related conditions [18, 19]. Preservation of the interspinous ligament combined with a skipped osteotomy approach facilitates the retrieval of posterior elements, while primarily serving to subsequent ease cadaver handling and transport. Once the procedure is finished, the abdominal organs are carefully placed back in their natural position, and the incision is closed in layers to ensure the integrity of the abdominal wall is restored.

### Post-Harvest specimen handling and evaluation

**Fig. 11:**
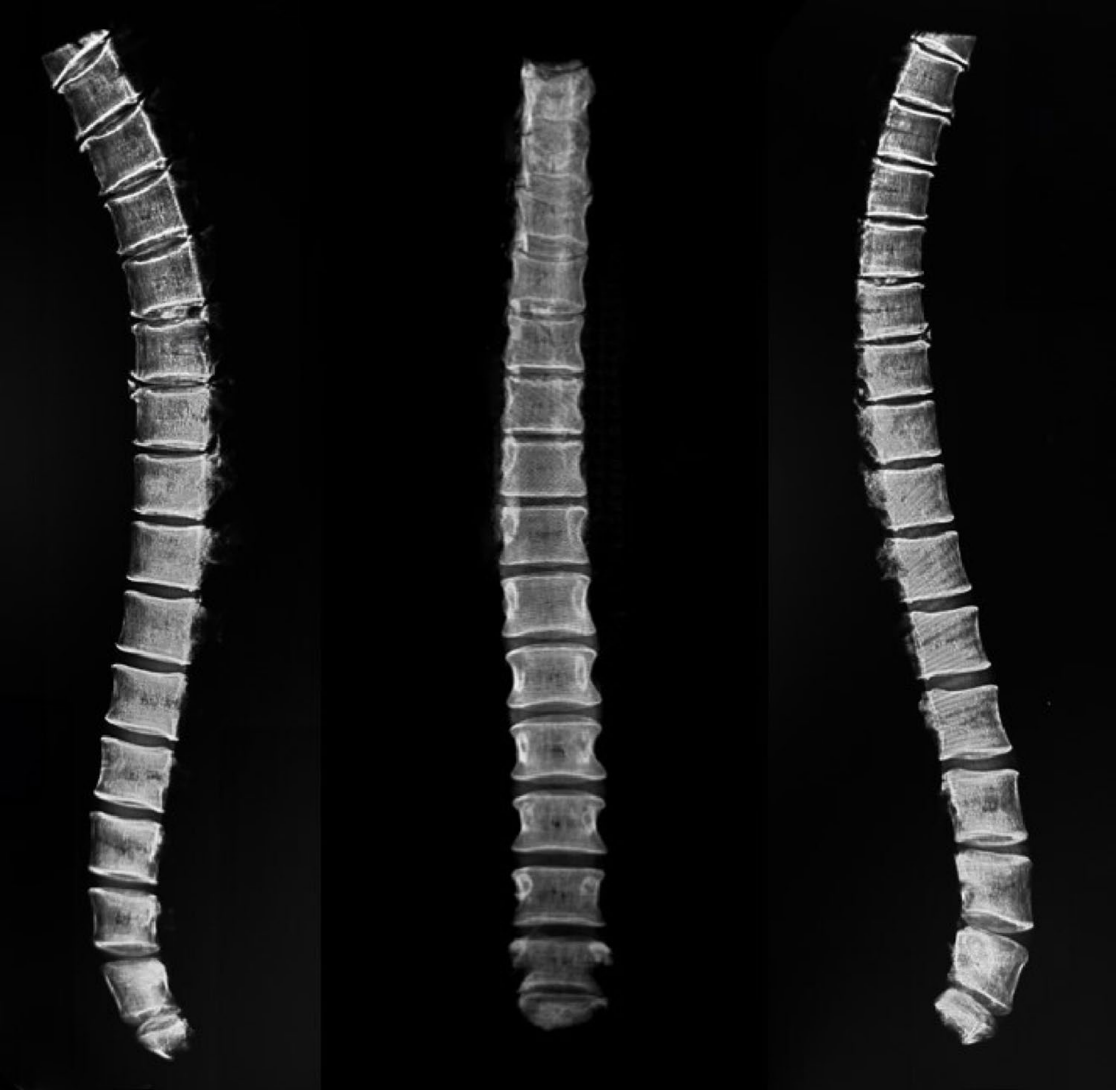
Anteroposterior and double-lateral radiographic assessment of spinal specimen

The harvested specimen is immediately placed on ice to preserve cellular viability and structural integrity of the tissues [20]. Subsequently, anteroposterior and double-lateral radiographs are performed to verify that the correct spinal segments have been harvested, to detect any potential bone damage during preparation, to assess the extent of spinal degeneration, and to identify any underlying pathologies (Fig. 11).

### Isolation protocols for human IVDs

#### Cell isolation from IVD tissue samples

**Fig. 12:**
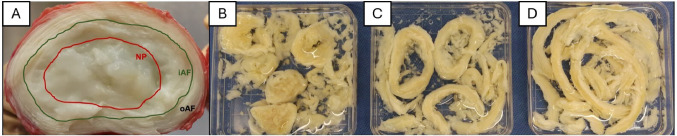
(**A**) Photomicrograph representing the intervertebral disc (IVD) and its three main tissues: nucleus pulposus (NP), inner annulus fibrosus (iAF), and outer annulus fibrosus (oAF). Images of isolated (**B**) NP, (C) iAF, and (D) oAF tissues

Human lumbar spines are harvested as described above, and discs are dissected as previously described [21–28]. The process begins approximately 2 h after the anterior spinal column is retrieved. The spine is rinsed with ethanol, and soft tissues are removed. Before cell isolation, IVDs are visually inspected for signs of degeneration, and photomicrographs are taken to assess the gross morphology of IVD midsagittal sections using the Thompson five-category grading scale of the human lumbar IVD [29]. Briefly, IVDs are carefully separated from the adjacent vertebral bodies using a scalpel, and the nucleus pulposus (NP), inner annulus fibrosus (iAF), and outer annulus fibrosus (oAF) are separated based on their distinct macroscopic morphologies, as illustrated in Fig. 12.

The tissues are separately minced and digested to enzymatically release the cells, as described in previous literature [21–28, 30]. Cell number and viability are assessed, 1 million cells are plated per T-75 culture flask, and maintained in a cell culture incubator until they reach 90% confluency [21–28, 30]. Passage 0 cells are cryopreserved and stored in liquid nitrogen for long-term storage. These cells are suitable for studying disc cell biology, IVD degeneration, extracellular matrix production, cellular senescence, inflammation, and regenerative approaches for intervertebral disc repair.

#### Intact IVDs isolation

As an alternative to cell isolation, an intact disc isolation approach may be employed, as previously described [26, 31–33]. This method preserves the native disc architecture, making it suitable for therapeutic studies involving bioreactor systems that maintain physiological loading conditions. The procedure allows for prolonged culture and functional assessment of the entire disc unit, maintaining the structural and biochemical integrity of the nucleus pulposus and annulus fibrosus.

### Isolation protocol for human facet joints

The cartilage from the facet joints is carefully dissected from the subchondral bone, cleaned, cut into small pieces, and the cells are enzymatically released as previously described [18, 19]. Briefly, chondrocyte number and viability are assessed, 1 million cells are plated per T-75 culture flask, and maintained in a cell culture incubator until they reach 90% confluency [18, 19]. Passage 0 cells are either used fresh or cryopreserved in liquid nitrogen for long-term storage. These cells are suitable for studying cartilage biology, cellular senescence, intervertebral joint degeneration, and potential regenerative strategies in spine-related disorders.

The remaining facet joint tissue is processed for osteoblast isolation as previously described [18, 19]. Briefly, subchondral bone is fragmented into small pieces using a rongeur, with cortical bone being primarily collected. The bone fragments are enzymatically digested to remove residual soft tissue. The digested bone chips are then placed in T-75 flasks and maintained in a cell culture incubator to allow osteoblasts to migrate out and adhere to the culture surface [18, 19]. Passage 0 cells are either used fresh or cryopreserved in liquid nitrogen for long-term storage. These cells are suitable for studying bone remodeling, spinal facet joint degeneration, osteogenic differentiation, and the molecular mechanisms underlying spine-related skeletal pathologies.

## Results

The protocol outlined has been successfully employed in over 340 spinal harvests since 2009. Following a brief learning curve, the surgical procedure requires approximately 30 min for completion. It is a safe, efficient, and reproducible method, suitable for adoption by both surgical teams and researchers. All harvested tissues retain viable cells throughout the procedure.

## Discussion

The present article introduces a surgical method for harvesting thoracolumbar spinal tissue, integrated seamlessly within the workflow employed during organ procurement procedures for transplantation purposes. It exemplifies a collaborative effort between surgical and laboratory teams aimed at advancing expertise and knowledge in translational spine research.

Unlike previously described harvesting techniques that require additional incisions on the cadaver, the present approach minimizes further disfigurement by limiting surgical access to the incision already used for organ procurement. Due to its simplicity and rapid execution, the technique may also be extended cranially to include the cervical spine in the tissue harvest, thereby broadening its applicability to the entire spinal column. Nevertheless, cranial extension of the surgical incision would result in a visible alteration of the cadaver, which may be evident during funeral preparations; for this reason, our team refrains from performing it.

The potential risk of retroviral transmission during tissue harvesting has previously been highlighted as a concern in organ retrieval procedures [34]. For example, the spinal harvesting technique described by Gorup et al. was carried out approximately three weeks postmortem—a delay that, while potentially reducing the risk of infection, significantly compromises tissue integrity due to advanced degradation. As a result, the harvested specimens are unsuitable for translational research aimed at investigating the cell biology of IVDs or facet joints [12]. In contrast, the present technique enables tissue retrieval within a much shorter postmortem interval. In addition, the fact that this procedure does not require the donor to be frozen and later thawed ensures the collection of sterile, well-preserved specimens that fully retain their biological and biochemical properties, thus supporting high-quality laboratory analyses. Furthermore, organ donors are subjected to a comprehensive infectious disease screening panel, a practice not commonly performed in cadaveric donors. Nevertheless, the potential risk of transmission must be acknowledged, and all appropriate personal protective measures, such as the use of multiple layers of gloves, fluid-shield masks, and ensuring vaccine coverage for major infectious diseases among the operating staff, are strongly recommended.

The harvested viable tissues and cells offer numerous downstream experimental opportunities, including bioreactor systems, genomic and transcriptomic analyses, qPCR, ELISA, cell culture and co-culture experiments, and histological studies. This unique repository has supported more than 95 publications in basic science, including studies on IVD biology, tissue engineering, and novel therapeutic strategies for low back pain.

Most spinal harvesting techniques described in the literature include a reconstruction phase to restore vertebral column continuity after tissue retrieval. For example, Yang et al. reported the use of wood and plaster to restore cervical bulk and rigidity for cosmetic purposes, while Yeh et al. employed a sharpened wooden rod inserted distally into the spinal canal and proximally through the foramen magnum to stabilize the donor’s neck [35, 36]. Although thoracolumbar spine harvesting typically places less emphasis on trunk reconstruction, as any instability in these areas is less noticeable, the technique described in this article does not require restorative intervention, since the posterior spinal elements are either preserved or harvested only partially. In this way, sufficient structural continuity of the trunk is maintained, preventing significant loss of consistency and avoiding the “destabilized” appearance that can occur when the spine is completely removed.

From a biomechanical standpoint, the described technique does not provide specimens suitable for studies that require preservation of the entire posterior complex and intact load-bearing continuity. For such research purposes, approaches involving en bloc removal of the whole spinal column may be more appropriate, although these procedures are technically demanding and usually associated with a considerable increase in operative time. In contrast, the present method offers an efficient and logistically simple option that yields fresh, biologically viable tissues particularly suited for cellular and translational investigations, while also facilitating cadaver handling and transport without the need for reconstruction.

## Conclusion

The present article introduces a human spine tissue harvesting technique that seamlessly integrates into the workflow employed in organ retrieval procedures for transplantation. The technique is safe, easy to perform, and has a short learning curve. Furthermore, it preserves sufficient structural continuity of the thoracolumbar spine to obviate the need for a reconstructive phase, thereby facilitating cadaver handling and transport. This procedure allows for the retrieval of fresh, sterile, and optimally preserved tissues, suitable for a wide range of experimental and translational research applications, although it may not be appropriate for biomechanical studies requiring intact posterior structures and full spinal stability.

## Data Availability

No datasets were generated or analysed during the current study.

## Abbreviations

AF: Annulus Fibrosus
AIS: Adolescent Idiopathic Scoliosis
DMEM: Dulbecco’s Modified Eagle Medium
DNA-seq: DNA Sequencing
DRGs: Dorsal Root Ganglia
ELISA: Enzyme–Linked Immunosorbent Assay
FBS: Fetal Bovine Serum
HBSS: Hank’s Buffered Saline Solution
HIV: Human Immunodeficiency Virus
IRB: Institutional Review Board
IVD: Intervertebral Disc
iAF: Inner Annulus Fibrosus
NP: Nucleus Pulposus
oAF: Outer Annulus Fibrosus
PBS: Phosphate–Buffered Saline
qPCR: Quantitative Polymerase Chain Reaction
RNA-seq: RNA Sequencing
